# A Cross-Sectional Study about the Associations between Physical Activity Level, Self-Perceived Health Perception and Mental Health in Informal Caregivers of Elderly or People with Chronic Conditions in Spain

**DOI:** 10.3390/ijerph19095320

**Published:** 2022-04-27

**Authors:** Ángel Denche-Zamorano, Laura Muñoz-Bermejo, Jorge Carlos-Vivas, María Mendoza-Muñoz, Juan Manuel Franco-García, Jorge Rojo-Ramos, Alejandro Vega-Muñoz, Nicolás Contreras-Barraza, Sabina Barrios-Fernandez

**Affiliations:** 1 Promoting a Healthy Society Research Group (PHeSO), Faculty of Sport Sciences, University of Extremadura, 10003 Caceres, Spain; andeza04@alumnos.unex.es (Á.D.-Z.); jorgecv@unex.es (J.C.-V.); 2 Social Impact and Innovation in Health (InHEALTH), University of Extremadura, 10003 Caceres, Spain; jorgerr@unex.es (J.R.-R.); sabinabarrios@unex.es (S.B.-F.); 3 Research Group on Physical and Health Literacy and Health-Related Quality of Life (PHYQOL), Faculty of Sport Sciences, University of Extremadura, 10003 Caceres, Spain; mamendozam@unex.es; 4 Departamento de Desporto e Saúde, Escola de Saúde e Desenvolvimento Humano, Universidade de Évora, 7004-516 Évora, Portugal; 5 Health Economy Motricity and Education (HEME), Faculty of Sport Sciences, University of Extremadura, 10003 Caceres, Spain; 6 Public Policy Observatory, Universidad Autónoma de Chile, Santiago 7500912, Chile; alejandro.vega@uautonoma.cl; 7 Facultad de Economía y Negocios, Universidad Andres Bello, Viña del Mar 2531015, Chile; nicolas.contreras@unab.cl

**Keywords:** caregivers, physical activity, mental health, physical health, self-perceived health, national survey

## Abstract

Providing informal care for older people, or people with chronic conditions, is associated with poorer physical and mental health and reduced quality of life. This task, in many cases, often relies on the women in the immediate family. Physical activity (PA) is a tool to enhance caregivers’ physical and mental health and their quality of life. Thus, this study aimed to analyse the associations between the physical activity level (PAL), self-perceived health (SPH) and mental health (SM) and its factors (positive coping, self-esteem, and stress) by conducting a cross-sectional study by using data from in the National Health Survey 2017 (ENSE 2017), the last one before the COVID-19 pandemic. The sample included 2225 caregivers (866 men and 1361 women). Descriptive analysis and non-parametric statistical tests, including chi-square, the Kruskal–Wallis test, the Mann–Whitney U test, and the Spearman’s rho correlation coefficient, were used. Dependence relationships were found between PAL and SPH and MH and their factors. The population groups that performed moderate or vigorous PA showed better results in both SPH and MH. Women scored worse than men in all the variables analysed. Hence, intense, or moderate PA practice may improve SPH and MH in Spanish informal caregivers, requiring the implementation of policies and programs considering the differences found between men and women in PAL, SPH, and MH.

## 1. Introduction

Population ageing in developed societies is causing an overload in social welfare and public health demand, increasing the need for policies to support people with dependency [1,2,3]. The United Nations (UN) estimates that the prevalence of people over 65 is expected to increase from 9% in 2019 to 16% in 2050, while the number of individuals over 80 is expected to triple from 143 million to 426 million [4]. Therefore, with the numbers of elderly growing so fast, the number of dependent people is increasing [5].

Formal caregivers are direct professionals who provide their care services for remuneration [6]. Informal care is the unpaid care provided to a person with whom they have a social relationship, such as family, relatives or friends [6,7]. Although, in many cases, families assume the major responsibility for care due to different socio-cultural and political factors, the trend is towards professionalising care [8]. Thus, the prevalence of informal caregivers is 11–17% in European countries [9,10] and around 20% both in the United States [11] and in low- and middle-income countries [12]. Nevertheless, about 80% of the informal caregivers are usually family members who often live with an older person. Although men are increasingly being incorporated into this role, women (usually wives and daughters) are usually those who adopt the responsibility of caregiving [13].

Perceived health status (SPH) indicates people’s overall perception of their health, including physical and psychological factors [14]. The SPH provides a summarised statement on how numerous aspects of health, both subjective and objective, fit together in the individual respondent’s perceptual framework [15]. The SPH has proved to be a reliable predictor of people’s health status, as it integrates objective knowledge of potential medical conditions with the interpretation of the individual’s physical and mental symptoms [16].

Caregivers of the elderly or people with chronic conditions usually experience a decline in physical and mental health [17,18,19]. Anxiety, depressive symptoms, higher levels of stress, lower levels of self-efficacy and subjective well-being are psychological and emotional consequences of the daily work of the informal caregiver [20,21]. These symptoms may be due to what is known as a subjective burden [22]. Prevalence studies have shown that 40.2% of elderly family caregivers have symptoms of anxiety and depression [21]. Moreover, caregivers have a poorer health perception [23], social support [10], physical activity (PA) [24] and, therefore, poorer health-related quality of life (HRQoL) [25,26,27].

A reduction in the burden of care has been linked to the caregiver’s sense of competence or self-efficacy and quality of life [28]. Among interventions to reduce the caregiver burden and negative consequences of care on physical and mental health, PA could be considered, and even performed together with the cared-for person [29]. Thus, the association between increased physical activity level (PAL) and improved physical and mental health (MH) has been well documented, including properly weight regulation [30], improved cardiorespiratory and muscular fitness, cardiometabolic health, bone health [31], mental health concerning depression [32], anxiety-related symptoms [33], and decreased pain [34], resulting in higher HRQoL levels [35,36]; these benefits have also been identified in caregivers [37,38,39].

This study hypothesizes that SPH and MH will be associated with PAL in the Spanish caregivers. The aim was to analyse the associations between PAL and SPH and MH in Spanish informal caregivers ranging 15–69 years of age. It sought differences between the sexes to characterise their needs appropriately, so that policies and programs adapted to their characteristics should be developed to improve their health, well-being and HRQoL. Additionally, it sought to provide a framework for comparing the status of these associations between pre- and post-pandemic COVID-19 periods.

## 2. Materials and Methods

### 2.1. Study Design and Ethical Concerns

A descriptive correlational study was conducted using The Adult Questionnaire from the ENSE 2017 [40,41,42].

Since the Regulation 2016/679 of the European Parliament and of the Council of 27 April 2016 on the protection of individuals with regard to the processing of personal data and on the free movement of personal data, and repealing Directive 95/46ECC [38] was published, public use files are not considered confidential. Thus, neither the application of data protection principles to anonymised information nor the approval of ethics committees, even for statistical or research purposes, is required [43].

### 2.2. Participants

Data were taken from the Spanish National Health Survey 2017 (ENSE 2017) [40]. This survey is developed every five years by the Ministry of Health, Consumption and Social Welfare and the National Institute of Statistics to study health-related factors to help them to assess and plan health policies.

**Figure 1:**
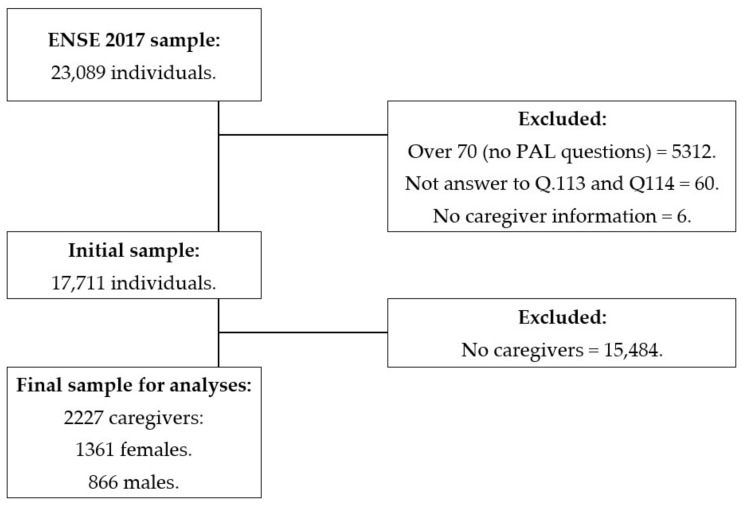
Chart outlining the study sample’s eligibility criteria.

The ENSE 2017 was carried out with 23,089 individuals (10,595 men and 12,494 women) residing in Spain and over 15 years old, using a random stratified three-phase sampling system [41]. For this study, firstly, those over 70 years were excluded as they were not asked about their PAL; those who did not answer the questions regarding their PAL; and those who did not confirm their status as a caregiver. Secondly, participants declaring not to be caregivers were excluded. Thus, the study sample was composed of 2227 caregivers (1361 females and 866 males) as shown in Figure 1.

### 2.3. Measures and Variables

During this research, the following variables were used and created.

#### 2.3.1. Extracted Variables

AGEa: Collected participants’ age, from 15–69 years old.

SEXOa Collected participants’ sex to characterise and group the sample by sex (male or female).

P133. Named “Cares” in the ENSE 2017. It collected the answers to the question: “Do you take care, at least once a week, of an elderly person or someone with a chronic ailment? Do not consider this whether it is part of your job”. Possible answers could be yes, no, do not know or no answer.

Q.135. Referred to as “Time spent caring” in the ENSE 2017. It collected the answers to the question: “In total, how many hours per week do you spend caring for this/these person(s)?”. Response options: less than 10 h per week, between 10–20 h, more than 20 h, and do not know/no answer.

G21. Referred to as ‘Self-perceived health’ in the ENSE 2017. It collected the answers to the question “In the last twelve months, would you say that your state of health has been very good, good, fair, poor, bad, very bad?” with the possible answers being: good, bad, very bad, do not know/no answer.

#### 2.3.2. Elaborated Variables

Mental health: built from the ENSE 3017 responses to questions from P47.1–P47.12 through the Spanish version of the Goldberg General Health Questionnaire (GHQ-12) [44,45]. Thus, the GHQ-12 is a psychological health tool composed of 12 items with four response options, ranging from 0 to 3 points, with 0 being the best health and 3 the worst, so that total scores vary between 0 and 36, with 0 being the best mental health and 36, the worst. This test has a high internal consistency (α = 0.86) [45].

- Successful coping (FI). The variable was derived from the GHQ-12. It was obtained with the sum of the responses to the items: Q.47.1, Q.47.3, Q.47.4, Q.47.7, Q.47.8 and Q.47.12. The score range was 0 and 18, with 0 being the most successful coping and 18 the worst. This factor has an external validity of 0.82 [46].
- Self-esteem (FII). The variable was derived from the GHQ-12. It was obtained from the sum of the responses to the items: Q.47.6, Q.47.9, Q.47.10 and Q.47.11. It presents scores between 0 and 12, with 0 being the highest self-esteem and 12, the lowest. This factor has an external validity of 0.70 [46]
- Stress (FIII). The variable was derived from the GHQ-12. It was obtained with the sum of the responses to the items: Q.47.2, Q.47.5 and Q.47.9. It presents scores between 0 and 9, with 0 being the least stressful and 9 the most stressful. This factor has an external validity of 0.75 [46].

Physical activity level (PAL): Three PALs were established based on the responses obtained in the ENSE 2017 to the PA-related questions on the International Physical Activity Questionnaire—Spanish version (IPAQ) [47]. This questionnaire asks about PA frequency, duration, and intensity the seven days before answering the survey. The intensity could be intense/vigorous, moderate, walking and sitting.

- Q113: “During the last seven days, how many days did you do vigorous physical activity? Vigorous activity requires great physical exertion and makes you breathe much harder than usual, such as heavy lifting, digging, aerobic exercise, or fast pedalling on a bicycle. Think only of those you performed for at least ten consecutive minutes”. Possible answers would be the number of days per week performed, none, do not know/no answer.
- Q.115: “During the last seven days, how many days did you do moderate physical activity? Please do not include walking. Moderate activities require moderate physical exertion that makes you breathe harder than usual, such as carrying light weights, cycling at a regular speed, or playing tennis doubles. Think only of those you did for at least ten consecutive minutes”. Possible answers would be the number of days per week performed, none, do not know/no answer.
- Q.117: “Now think about how much time you spent walking in the last seven days. This includes walking at work, home, and from one place to another, or walking for sport, exercise, or pleasure. Think only of those occasions when you walked for at least ten consecutive minutes”. Possible answers would be the number of days per week, none, do not know/no answer.

Individuals were considered “Inactive” when they responded “no days per week” to items Q.113, Q.115 and Q.117, who neither walked on at least one day per week for more than 10 min at a time, nor performed moderate and/or intense PA on at least one day per week for more than ten consecutive minutes. Participants were considered “Walkers” when they answered Q.113 and Q.115 with at least one day a week and more than ten consecutive minutes to item Q.117. Individuals were considered “Active” when they responded to Q.113 and Q.115 with at least one day a week for more than ten consecutive minutes.

### 2.4. Statistical Analysis

Data distribution was analysed using the Kolmogorov–Smirnov test. Non-parametric tests were carried out. A descriptive analysis to characterise the sample was performed, presenting the continuous variables according to the central values: median and interquartile range, with mean and standard deviation as additional information; and the ordinal variables, according to absolute and relative frequency.

Mann–Whitney U (for continuous variables) and Chi-square (for ordinal variables) tests were carried out to analyse potential differences between sexes and groups. Mann–Whitney U was used to assess PAL between-group differences. The Kruskal–Wallis test was used to find differences at baseline between PAL and GHQ-12 derived continuous variables. Finally, a correlation study using Spearman’s Rho was undertaken to analyse the associations between PAL, SPH, MH and their different factors.

## 3. Results

The Kolmogorov–Smirnov test did not provide sufficient evidence to assume that the data of the variables followed a normal distribution, so it was assumed that they followed a non-normal distribution (*p* < 0.001).

According to Table 1, with the median of years 47 years and interquartile range of 21 for both men and women, and not finding differences between sexes, it was assumed that the caregiver sex did not determine the differences found during this study. A dependency relationship was obtained between caregivers’ prevalence and sex, with a higher proportion of female caregivers than men.

Once the eligibility criteria were applied, a median age of 52 years was obtained, with no differences between the sexes. Dependency relations were found between the amount of time spent caring, SPH, MH and its factors (FI, FII and FIII), and the sex of the caregivers (Table 2).

Table 3 shows the associations between the PAL and SPH in informal caregivers according to the ENSE 2017, finding dependence relationships between them.

**Figure 2:**
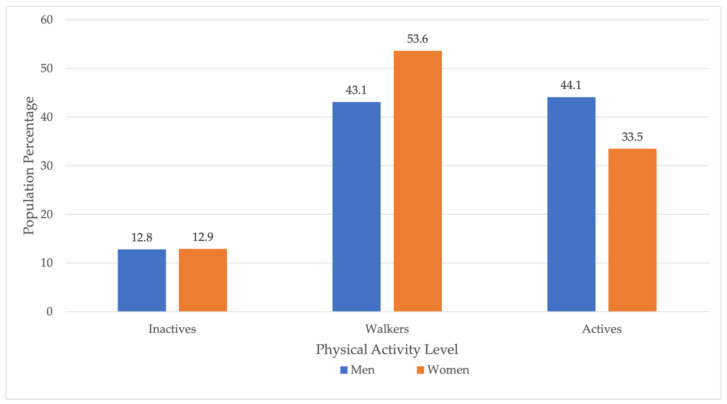
Comparison of physical activity level by sex (men and women).

Figure 2 illustrates the PAL differences, considering inactives, walkers and actives. regarding sex (men and women).

**Figure 3:**
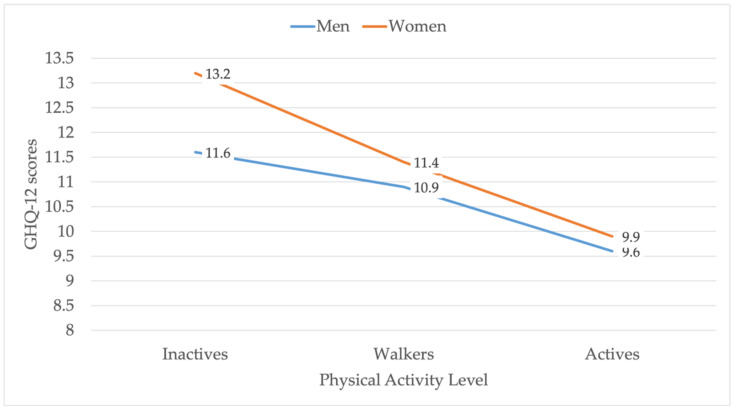
Comparison of mean mental health scores according to physical activity level, differentiated by sex (men and women). GHQ-12: Goldberg General Health Questionnaire.

Table 4 and Figure 3 display the associations between male and female informal caregivers’ PAL and MH and its subscales through the GHQ-12. The Kruskal–Wallis test evaluated significant differences in MH and its subscales, using PAL as factors, finding significant differences (*p* < 0.001 in MH, FI and FII. *p* = 0.002 in FIII). These tables also show differences in MH and its subscales between PAL groups, according to the Mann–Whitney U test.

Finally, weak but significant correlations were found between PAL and SPH (0.173), MH (−0.210), and its factors: FI Successful coping (−0.178), FII Self-esteem (−0.196) and FIII Stress (−0.167) using Spearman’s Rho.

## 4. Discussion

### 4.1. Main Findings and Theoretical Implications

The main finding of this study was a dependency relationship between PAL and SPH and MH in Spanish informal caregivers aged 15–69 years during the period before the COVID-19 pandemic, confirming our hypothesis. Thus, higher PAL was associated with better SPH and MH status. Walking improved SPH compared to inactivity, although activity at moderate and/or vigorous intensity was an even greater improvement; MH, especially self-esteem, improved as PAL increased.

The prevalence of informal caregivers caring for the elderly or people with chronic conditions is approximately 12.6% in Spain. There exists a gender gap mostly due to gender roles in which traditionally, women have been in charge of caring for dependent family members [48]. Moreover, when women are the caregivers, they spend more time caring, so the amount of time spent caring seems to be sex-dependent. Spanish informal caregivers with more than 20 h of caregiving have a gender gap of 9.5 percentage points, 43.9% in women versus 34.4% in men, in line with previous analyses of the Spanish population [49]. Female caregivers’ SPH was worse than male ones, showing a sex-dependent relationship in this variable. There were differences between the proportions in both sexes, with a lower proportion of women reporting a “very good” SPH than the male ratios. Moreover, MH, including its three factors, was also worse in female caregivers. Among the potential factors explaining these results could be the greater amount of time spent caring, greater involvement in caregiving, or the lack of social support found in women. Nevertheless, male caregivers showed greater attention to their health [50,51] and better MH, which may be due to better-perceived self-efficacy and self-confidence and a lower perception of caregiving overload [52].

Regarding PA, 55.9% of male caregivers and 66.5% of female caregivers did not perform vigorous or moderate PA and so did not reach the World Health Organisation’s PA recommendations, which recommend at least 75 min of vigorous PA per week, 150 min of moderate PA, or equivalent to ensure health [31]. Another finding was that women’s proportion of “walkers” (53.6%) was ten percentage points higher than men’s (43.1%); it is still insufficient, although healthier than “inactive”, in line with other studies [53,54]. These results could be related to the female caregivers’ poorer SPH relative to men’s (66.6% vs. 72% positive SPH). The relationship between PAL and physical and mental health is well documented in the general population [35,36,55,56]. This study found a dependency relationship between the PAL level and caregivers’ SPH. Thus, “active” men who reported experiencing “good” or “very good” SPH represented 81.4% compared to 61.3% of “inactive” men; “active” women who reported “good” or “very good” SPH were 75.2% compared to 49.2% of “inactive” women. In this line, several studies highlight a need to focus on PA programs from a gender perspective, as women tend to report a worse SPH [57,58]. Those caregivers who were physically active presented better MH and improved successful coping, self-esteem, and stress. Thus, there is a two-point difference between active and inactive male caregivers and a 3.24 difference in female caregivers. This association has also been observed in the general population [59,60,61] and informal caregivers [37,48]. In this sense, caregivers performing moderate PA improved their perception of self-efficacy, positive affect, stress levels and sleep [62,63], while the report of depressive symptoms, hence psychological distress, decreased [64]. Having PA in caregivers appears to positively impact the subjective burden of caregiving [61,65,66].

### 4.2. Practical Implications

The results suggests that PA programs should be a suitable intervention to be offered to enhance the mental well-being of caregivers of the elderly or people with chronic conditions’. Moreover, the results also confirmed the importance of (1) supporting informal caregivers in the caregiving process, allowing them to achieve adequate occupational balance, health and HRQoL, (2) being able to perform a PAL compatible with good levels of physical and mental health, (3) considering a gender perspective, as socio-cultural aspects may be influencing both the provision of care and the decision to perform it, and (4) taking into account the gender perspective, as socio-cultural aspects may influence it both in the provision of care and in the decision to perform or not PA, as well as the type of activity chosen. This is in line with the Sustainable Development Goals, in this case, with goals 3 “Health and well-being” and 10 “Reducing inequalities” [67].

Another interesting application is that this study was conducted with data from the ENSE 2017, the last one before the pandemic, so it will offer a framework to compare these findings and their potential variations after the confinement period and the removal of the leading sanitary measures around the COVID-19. In this regard, the importance of strengthening mental wellbeing policies for caregivers should be highlighted, as they are a fundamental pillar in the care network for dependent people.

### 4.3. Limitations and Future Lines

The ENSE provides representative data on caregivers’ health and the relationship between socio-demographic characteristics, SPH, PA and MH. However, this study also has limitations. (1) Differences between moderate and intense PA were not analysed, so the relationships between activity intensity and mental well-being could not be established, which should be considered in future research. (2) The GHQ-12 is a screening tool, more sensitive than specific, which may lead to an overestimation of MH problems, especially in older people. (3) As this was a cross-sectional study, it was not possible to establish cause-effect relationships. (4) The lack of access to the participants’ medical records means that the study does not concretely investigate mental disorders. It would also be interesting to add an objective, physiological and follow-up data, and to carry out a 24-h compositional analysis, including devices to quantify PA or intensity, or other measures that could overcome the limitations of studies based on surveys and the subjective perception of the participants.

## 5. Conclusions

The SPH and MH in informal caregivers aged from 15 to 69 were related to PAL. The results suggest that caregivers who performed PA showed better SPH and mental well-being in the three factors: successful coping, self-esteem, and reduced stress. Increasing PAL in Spanish informal caregivers, including intense and/or moderate PA, could improve their SPH and MH. However, walking could be an alternative to consider, without achieving such good results.

The MH policies and PA programs aimed at informal caregivers should include a gender perspective because care tasks have a greater impact on women.

## Figures and Tables

**Table 1:** The age and number of caregivers according to the Spanish National Health Survey 2017.

Age (Years)	Total = 17,711	Men = 8486	Women = 9225	*p*-Value from the Mann–Whitney U Test
Median (IQR)	47 (21)	47 (21)	47 (21)	0.329
Caregivers	Total = 17,711	Men = 8486 *n* (%)	Women = 9225 *n* (%)	*p*-Value from the Chi-Square Test
Yes	2227 (12.6)	866 (10.2) ^a^	1361 (14.8) ^b^	<0.001
No	15,484 (87.4)	7620 (89.8) ^a^	7864 (85.2) ^b^	

IQR: interquartile range; *n*: number of participants; %: percentage; Yes: cares for elderly or people with chronic conditions at least one day a week out of work; No: does not care for elderly or people with chronic conditions at least one day a week out of work; ^ab^: Different subscripts denote that column proportions differ at a significance level < 0.05.

**Table 2:** Informal age of the caregivers, amount of time caring, self-perceived health, mental health, and their subscales according to the Spanish National Health Survey 2017.

Age (Years)	Total = 2227	Men = 866 *n* (%)	Women = 1361 *n* (%)	*p*-Value from the Chi-Square Test
Median (IQR)	52 (15)	52 (15)	52 (14)	0.272
Caring Amount of Time (hours)	Total = 2220	Men = 863 *n* (%)	Women = 1357 *n* (%)	
Less than 10	870 (39.0)	383 (44.4) ^a^	485 (35.8) ^b^	<0.001
Between 10–20	461 (20.7)	183 (21.2) ^a^	276 (20.3) ^a^	
More than 20	897 (40.3)	297 (34.4) ^a^	596 (43.9) ^b^	
Self-Perceived Health	Total = 2227	Men = 866 *n* (%)	Women = 1366 *n* (%)	
Very good	300 (13.5)	138 (15.9) ^a^	162 (11.9) ^b^	<0.001
Good	1231 (55.3)	486 (56.1) ^a^	745 (54.7) ^a^	
Fair	530 (23.8)	181 (20.9) ^a^	349 (25.6) ^b^	
Bad	137 (6.2)	52 (6.0) ^a^	85 (6.2) ^a^	
Very Bad	29 (1.3)	9 (1.0) ^a^	20 (1.5) ^a^	
Mental Health (GHQ-12)	Total = 2215	Men = 863	Women = 1352	*p*-Value from the Mann–Whitney U test
Median (IQR)	10 (4)	10 (5)	10 (4)	<0.001
Mean (SD)	10.9 (4.8)	10.4 (4.7)	11.1 (4.9)	
FI. Successful coping	Total = 2215	Men = 863	Women = 1352	
Median (IQR)	6 (0)	6 (0)	6 (0)	0.506
Mean (SD)	6.3 (1.8)	6.3 (1.7)	6.4 (1.8)	
FII. Self-esteem	Total = 2215	Men = 863	Women = 1352	
Median (IQR)	2 (4)	2 (4)	2 (4)	<0.001
Mean (SD)	2.4 (2.4)	2.2 (2.3)	2.5 (2.4)	
FIII. Stress	Total = 2215	Men = 866	Women = 1357	
Median (IQR)	3 (3)	3 (3)	3 (2)	<0.001
Mean (SD)	3.0 (2.1)	2.6 (2.1)	3.1 (2.1)	

IQR: interquartile range; SD: standard deviation; *n*: number of participants; %: percentage; amount of time caring: cares for elderly or people with chronic conditions at least one day a week out of work; GHQ-12: Goldberg’s General Health Questionnaire, with scores between 0 and 36, 0 being the best mental health and 36 the worst; F: factor; FI Successful coping: scores from 0 to 18; FII Self-esteem: scores from 0 to 9; FIII Stress: scores from 0 to 9; ^ab^: different subscripts assume that column proportions differ at a significance level <0.05.

**Table 3:** Associations between the physical activity level and self-perceived health in informal caregivers according to the Spanish National Health Survey 2017.

Physical Activity Level							
Sex	Inactives *n* (%)		Walkers *n* (%)		Actives *n* (%)		*p*-Value from the Chi-Square Test
Men	111 ^a^	(12.8)	373 ^a^	(43.1)	382 ^a^	(44.1)	<0.001
Women	175 ^a^	(12.9)	730 ^b^	(53.6)	456 ^b^	(33.5)	
Total	286	(12.8)	1103	(49.5)	838	(37.6)	
Men							
SPH	Inactives *n* (%)		Walkers *n* (%)		Actives *n* (%)		
Very good	11 ^a^	(9.9)	44 ^a^	(11.8)	83 ^a^	(21.7)	<0.001
Good	57 ^a^	(51.4)	201 ^a^	(53.9)	228 ^a^	(59.7)	
Fair	30 ^a^	(27.0)	92 ^a^	(27.4)	59 ^b^	(15.4)	
Bad	12 ^a^	(10.8)	30 ^a^	(8.0)	10 ^b^	(2.6)	
Very bad	1 ^a^	(0.9)	6 ^a^	(1.6)	2 ^a^	(0.5)	
Women							
SPH	Inactives *n* (%)		Walkers *n* (%)		Actives *n* (%)		
Very good	11 ^a^	(6.3)	85 ^ab^	(11.6)	66 ^b^	(14.5)	<0.001
Good	75 ^a^	(42.9)	393 ^b^	(53.8)	277 ^b^	(60.7)	
Fair	57 ^a^	(32.6)	195 ^ab^	(26.7)	97 ^b^	(21.3)	
Bad	25 ^a^	(14.3)	44 ^b^	(6.0)	16 ^b^	(3.5)	
Very bad	7 ^a^	(4.0)	13 ^a^	(1.8)	0 ^b^	(0.0)	

Inactive: reported not engaging in vigorous and/or moderate physical activity or walking at least one day a week for more than 10 min; walkers: reported not engaging in vigorous and/or moderate physical activity at least one day a week for more than 10 min but walking at least one day a week for more than 10 min; active: reported not engaging in vigorous and/or moderate physical activity at least one day a week for more than 10 min; ^ab^: each letter of the subscript denotes a subset of the physical activity level categories, whose column proportions do not differ from each other at level <0.05.

**Table 4:** Associations between the (a) male and (b) female informal caregivers’ physical activity level and mental health and its subscales through the Goldberg General Health questionnaire according to the Spanish National Health Survey 2017.

(a)							
PAL		GHQ-12	PAL	Med. Diff.	Mean Diff.	*p* *	*p* **
Inactives	Med. (IQR)	10 (5)	Walkers	0	0.7	<0.001	0.667
	Mean (SD)	11.6 (6.3)	Actives	1	2.0		0.003
Walkers	Med. (IQR)	10 (5)	Inactives	0	−0.7	<0.001	0.667
	Mean (SD)	10.9 (4.6)	Actives	1	1.3		<0.001
Actives	Med. (IQR)	9 (5)	Inactives	−1	−2.0	<0.001	0.003
	Mean (SD)	9.6 (4.1)	Walkers	−1	−1.3		<0.001
PAL		FI	PAL	Med. Diff.	Mean Diff.	*p* *	*p* **
Inactives	Med. (IQR)	6 (1)	Walkers	0	0.4	<0.001	0.152
	Mean (SD)	6.9 (2.4)	Actives	0	0.9		<0.001
Walkers	Med. (IQR)	6 (0)	Inactives	0	−0.4	<0.001	0.152
	Mean (SD)	6.5 (3.0)	Actives	0	0.5		<0.001
Actives	Med. (IQR)	6 (0)	Inactives	0	−0.9	<0.001	<0.001
	Mean (SD)	6.0 (1.4)	Walkers	0	−0.5		<0.001
PAL		FII	PAL	Med. Diff.	Mean Diff.	*p* *	*p* **
Inactives	Med. (IQR)	2 (4)	Walkers	0	0.3	<0.001	0.820
	Mean (SD)	2.7 (2.9)	Actives	1	0.9		0.011
Walkers	Med. (IQR)	2 (4)	Inactives	0	−0.3	<0.001	0.820
	Mean (SD)	2.4 (2.2)	Actives	−1	0.6		<0.001
Actives	Med. (IQR)	1 (3)	Inactives	−1	−0.3	<0.001	0.011
	Mean (SD)	1.8 (2.2)	Walkers	−1	−0.6		<0.001
PAL		FIII	PAL	Med. Diff.	Mean Diff.	*p* *	*p* **
Inactives	Med. (IQR)	3 (3)	Walkers	0	0.0	<0.002	0.886
	Mean (SD)	2.9 (2.6)	Actives	1	0.5		0.041
Walkers	Med. (IQR)	3 (3)	Inactives	0	0.0	<0.002	0.886
	Mean (SD)	2.9 (2.1)	Actives	1	0.5		0.001
Actives	Med. (IQR)	2 (2)	Inactives	−1	−0.5	<0.002	0.041
	Mean (SD)	2.4 (2.0)	Walkers	−1	−0.5		0.001
(b)							
PAL		GHQ-12	PAL	Med. Diff.	Mean Diff.	*p* *	*p* **
Inactives	Med. (IQR)	12 (7)	Walkers	1	1.74	<0.001	<0.001
	Mean (SD)	13.15 (5.89)	Actives	3	3.24		<0.001
Walkers	Med. (IQR)	11 (5)	Inactives	−1	−1.74	<0.001	<0.001
	Mean (SD)	11.41 (4.89)	Actives	2	1.5		<0.001
Actives	Med. (IQR)	9 (5)	Inactives	−3	−3.24	<0.001	<0.001
	Mean (SD)	9.91 (3.96)	Walkers	−2	−1.5		<0.001
PAL		FI	PAL	Med. Diff.	Mean Diff.	*p* *	*p* **
Inactives	Med. (IQR)	6 (2)	Walkers	0	0.61	<0.001	<0.001
	Mean (SD)	7.04 (2.30)	Actives	0	1.06		<0.001
Walkers	Med. (IQR)	6 (1)	Inactives	0	−0.61	<0.001	<0.001
	Mean (SD)	6.43 (1.86)	Actives	0	0.45		<0.001
Actives	Med. (IQR)	6 (0)	Inactives	0	−1.06	<0.001	<0.001
	Mean (SD)	5.98 (1.28)	Walkers	0	−0.45		<0.001
PAL		FII	PAI	Med. Diff.	Mean Diff.	*p* *	*p* **
Inactives	Med. (IQR)	4 (4)	Walkers	2	1.05	<0.001	<0.001
	Mean (SD)	3.61	Actives	3	1.66		<0.001
Walkers	Med. (IQR)	2 (3)	Inactives	−2	−1.05	<0.001	<0.001
	Mean (SD)	2.56 (2.38)	Actives	1	0.61		<0.001
Actives	Med. (IQR)	1 (3)	Inactives	−3	−1.66	<0.001	<0.001
	Mean (SD)	1.95 (2.10)	Walkers	−1	−0.61		<0.001
PAL		FIII	PAI	Med. Diff.	Mean Diff.	*p* *	*p* **
Inactives	Med. (IQR)	3 (2)	Walkers	0	0.30	<0.001	<0.001
	Mean (SD)	3.61 (2.1)	Actives	0	0.93		<0.001
Walkers	Med. (IQR)	3 (3)	Inactives	0	−0.30	<0.001	<0.001
	Mean (SD)	3.31 (2.08)	Actives	0	0.64		<0.001
Actives	Med. (IQR)	3 (3)	Inactives	0	−0.93	<0.001	<0.001
	Mean (SD)	2.68 (2.09)	Walkers	0	−0.64		<0.001

PAL: level of physical activity; inactive: reported not engaging in vigorous and/or moderate physical activity or walking at least one day a week for more than 10 min; walkers: reported not engaging in vigorous and/or moderate physical activity at least one day a week for more than 10 consecutive minutes, although they did walk at least one day a week for more than 10 min; actives: reported engaging in vigorous and/or moderate physical activity at least one day a week for more than 10 min; GHQ-12: Goldberg’s General Health Questionnaire, scores between 0–36, 0 being the best mental health and 36 the worst; FI: Factor I Successful Coping, scores between 0–18, 0 being the best coping and 18 the worst; FII: Factor II Self-Esteem, scores between 0–9, 0 being the best self-esteem and 9 the worst; FIII: Factor III Stress, scores between 0–9, 0 being the least stressful and 9 the most stressful; FIII: Factor III Stress, scores between 0–9, 0 being the least stressful and 9 the most stressful; Med Diff: differences between the mental health medians for each physical activity level; Mean Diff: differences between the mental health means for each physical activity level; *p* *: global Kruskal–Wallis for a *p* = 0.05 using as response mental health and its factors according to the GHQ-12, and as a factor the physical activity level; *p* **: Mann–Whitney U test derived from the comparison between the mental health medians and its factors for each physical activity level. IQR: interquartile range; SD: standard deviation.

## Data Availability

Data used were obtained from public use files, available on the Spanish Ministry of Health, Consumer Affairs, and Social Welfare website: https://www.mscbs.gob.es/estadEstudios/estadisticas/encuestaNacional/encuesta2017.htm (accessed on 14 January 2022). Additional datasets will be available under reasonable request.
